# Esophageal perforation and obstruction during retrieval of gastrointestinal stromal tumor, with final retrieval using laryngoscope

**DOI:** 10.1055/a-2643-8931

**Published:** 2025-08-08

**Authors:** Suhua Wu, Song He, Yalan Song, Chengkai Gao, Chao Deng

**Affiliations:** 1 585250 Department of Gastroenterology, The Second Affiliated Hospital of Chongqing Medical University, Chongqing, China; 2 585250 Department of Otolaryngology, The Second Affiliated Hospital of Chongqing Medical University, Chongqing, China

A 70-year-old female patient was found to have a 4.0 cm × 3.6 cm submucosal tumor in the gastric fundus ( Fig. 1 **a** ). Enhanced computed tomography and endoscopic ultrasonography were performed, and the lesion was considered to be a gastrointestinal stromal tumor (GIST). Endoscopic full-thickness resection was performed, achieving complete tumor excision and gastric wall closure. Our team had previously reported that a balloon-assisted method was used to completely remove a GIST of a similar size 1 . With the assistance of the balloon, the tumor mass successfully passed through the cardia but got stuck at the esophageal entrance. Due to the extreme hardness of the tumor mass, it could not be deformed to pass through the esophageal entrance even with the assistance of the balloon. Forceful traction caused esophageal rupture, and the tumor mass was embedded in the rupture site, compressing the hypopharyngeal posterior wall. When attempting to push the tumor mass back into the stomach, it rotated through the rupture site, and the attempt failed.

**Fig. 1:**
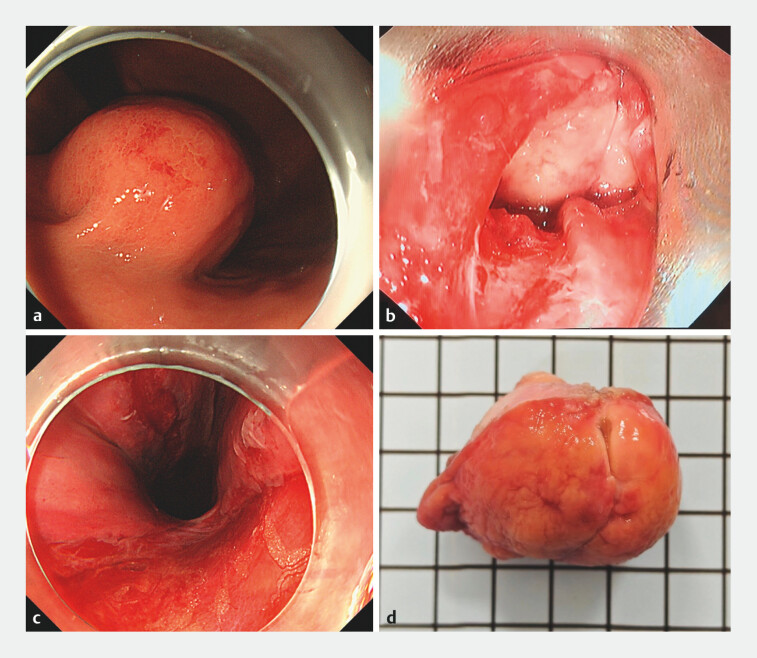
Endoscopic en-bloc retrieval of large gastric gastrointestinal stromal tumor specimen with the assistance of laryngoscope. **a** A 4.0-cm × 3.6-cm gastric gastrointestinal stromal tumor was located in the gastric fundus. **b** A rigid laryngoscope was used under the guidance of a second gastroscope to lift the hypopharynx, revealing that the tumor got stuck at the rupture site of the esophageal entrance. **c** A full-thickness tear approximately 5 cm in length was visible, extending from the esophageal entrance to the posterior wall of the hypopharynx. **d** The specimen was complete with a volume of 4.0 cm × 3.6 cm × 3.5 cm.

Otolaryngologists were invited to use a rigid laryngoscope to lift the hypopharynx, which exerted much greater force than balloon dilation ( Fig. 1 **b** ). The tumor mass successfully disengaged from the rupture site and was completely removed along the laryngoscope. The tear at the esophageal entrance extending to the posterior wall of the hypopharynx was approximately 5 cm long and was completely closed with 15 clips ( Fig. 1 **c** ). The patient was discharged smoothly on the fifth day. The final pathological examination revealed a low-risk GIST with dimensions of 4.0 cm × 3.6 cm × 3.5 cm ( Fig. 1 **d** ). This case highlights the value of multidisciplinary collaboration and the flexible use of different instruments in addressing unexpected difficulties ( Video 1 ).

The assistance of laryngoscope for en-bloc retrieval of large gastric gastrointestinal stromal tumor specimen.Video 1

Endoscopy_UCTN_Code_CPL_1AH_2AZ_3AD

## Notes

Endoscopy E-Videos https://eref.thieme.de/e-videos
*E-Videos* is an open access online section of the journal *Endoscopy* , reporting on interesting cases and new techniques in gastroenterological endoscopy. All papers include a high-quality video and are published with a Creative Commons CC-BY license. Endoscopy E-Videos qualify for HINARI discounts and waivers and eligibility is automatically checked during the submission process. We grant 100% waivers to articles whose corresponding authors are based in Group A countries and 50% waivers to those who are based in Group B countries as classified by Research4Life (see: https://www.research4life.org/access/eligibility/ ). This section has its own submission website at https://mc.manuscriptcentral.com/e-videos .
